# Metabolomics - a useful tool for prediction of protein production and processing?

**DOI:** 10.1186/1753-6561-5-S1-P80

**Published:** 2011-11-22

**Authors:** Jennifer Kronthaler, Timo Schmidberger, Christine Heel

**Affiliations:** 1 University of Innsbruck, Innsbruck, Austria; 2 Sandoz Biopharmaceuticals, Langkampfen, Austria

## Introduction

Although CHO cells are widely used as hosts for recombinant protein production, still only little is known regarding the interaction of metabolic alterations and protein production and processing. Therefore, a more sophisticated look into the cellular metabolism might lead to a more efficient use of the production medium resulting in high quantities of the protein with the desired product quality. For intracellular metabolite quantification the sample preparation including quenching of cells is a very critical step strongly affecting subsequent results. A simple and straightforward protocol, not needing special equipment, was investigated focusing on the efficiency of stopping metabolic activities and the potential metabolic leakage due to losses in membrane integrity. In a next attempt, the comparison between the producer cell line and the corresponding mock cell line using targeted mass spectrometry approaches for intra- and extracellular metabolite quantification was performed. The influence of the increased protein production capacity and the need of a complex glycosylation machinery on metabolite profiles was assessed. In order to find a link between the determined output parameters, detailed data evaluation including multivariate data analysis tools was implemented.

## Materials and methods

CHO producer cell line as well as corresponding mock cell line (transfected with an empty plasmid) were cultivated as a fed-batch process in serum-free, chemically defined in-house medium, comprising insulin. For the experiments 15L bioreactors were used. Conditions were 36,5°C and ph 6,9. Sampling for subsequent metabolite quantification (intra- as well as extracellular) took place at various time points throughout cultivation.

Targeted metabolomic analysis was performed for cell culture supernatants as well as for cell lysates using freeze/thaw cycles after resuspension in phosphate buffer for extraction. For the quantification of amino acids, hexose and biogenic amines commercially available AbsoluteIDQ KIT plates were used. This fully automated assay was based on PITC (phenylisothiocyanate)-derivatization in the presence of internal standards followed by LC-MS/MS detection using a AB SCIEX 4000 QTrap™ mass spectrometer with electrospray ionization [1]. For the quantitative analysis of energy metabolism intermediates (glycolysis, citrate cycle, urea cycle), a hydrophilic interaction liquid chromatography (HILIC)-ESI-MS/MS method in highly selective negative MRM detection mode was used. Intracellular amounts of nucleotides and nucleotide sugars were analyzed by liquid chromatography-electrospray ionization-mass spectrometry on surface-conditioned porous graphitic carbon as described by Pabst et al. [2].

## Results

The comparison between a CHO producer cell line and the corresponding mock cell line enabled the assessment of increased protein production capacity and the need of a complex glycosylation machinery influencing metabolite profiles. Multivariate data analysis using a PLS (partial least square) model on all quantified compounds showed similar progress throughout fermentation for all four cultivations (producer and mock in duplicates). Changes between different sampling points, which represent different stages of cultivation showed the strongest impact. Thus, differences between tested cell lines have to be evaluated for each phase individually as no overall alteration is detectable.

**Figure 1:**
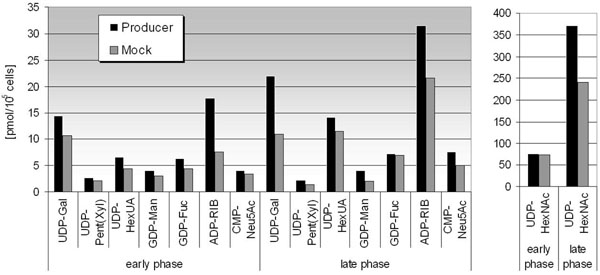
Intracellular nucleotide sugar concentrations

Nucleotide sugars which play important key roles within the mammalian glycosylation pathway showed the major variance between producer and mock cell line, as shown in figure 1: Early phase represents data within exponential phase (up to and including day 7) whereas late phase shows averaged data of day 10 and 12. The increased glycosylation processing in producer cells seemed to lead to higher intracellular concentrations of required precursors due to increased stimulation of the overall glycosylation machinery. Otherwise, an increased consumption by producer cells should have led to lower concentrations or even to limiting bottlenecks. The increased amounts of intracellular nucleotide sugars at late stage for both cell lines were evident, but an adequate explanation with respect to cell- and process knowledge is still missing.

Moreover, within exponential phase (sampling on day 3-7) specific consumption/production rates of divers metabolites were different between producer and mock cell line. Mostly, amino acids were affected. Mock cells showed an increased energy demand reflected by higher specific consumption rates. Thus, especially the slightly higher growth rate of mock cells seemed to be of relevance resulting in a differentiation between proliferating and producing cells. However, detailed investigation of impacts of involved pathways on recombinant protein production and processing is still ongoing.

## Conclusion and outlook

Within this study the application of detailed metabolite data to mammalian process development was evaluated preliminarily. Just marginal differences between producer and mock cells were detectable. However, further pathway-based investigations are still ongoing.
